# Acute Stress Response of Sheep to Shearing Procedures: Dynamic Change of Cortisol Concentration and Protein Electrophoretic Pattern

**DOI:** 10.3390/ani12070862

**Published:** 2022-03-29

**Authors:** Francesca Arfuso, Francesco Fazio, Lucas Chikhi, Guillaume Aymond, Giuseppe Piccione, Claudia Giannetto

**Affiliations:** 1 Department of Veterinary Sciences, University of Messina, 98168 Messina, Italy; farfuso@unime.it (F.A.); ffazio@unime.it (F.F.); claudia.giannetto1@unime.it (C.G.); 2 École Nationale Vétérinaire de Toulouse, Chemin des Capelles, 31300 Toulouse, France; chikhilucas@gmail.com (L.C.); guillaume.aymond_18@envt.fr (G.A.)

**Keywords:** acute stress, sheep, shearing, stress response, serum protein fractions, electrophoresis

## Abstract

**Simple Summary:**

Farm animals are daily subjected to a wide range of abiotic stressors. Shearing, one practice of routine management procedures, is applied periodically to harvest sheep’s wool. During shearing, short-term acute stress is likely to occur. This study aimed to evaluate the change in hematological parameters, serum cortisol concentration and serum protein electrophoretic pattern in Comisana sheep following shearing procedures. The findings obtained in the current study suggest a linkage between the endocrine and immune systems and an acute phase response during the shearing procedure.

**Abstract:**

The current study aimed to investigate the influence of acute stress by shearing procedures on hematological parameters, serum cortisol concentration and serum protein electrophoretic pattern in Comisana sheep. A total of 20 not pregnant and not lactating adult ewes, aged 3–4 years old and with a mean bodyweight of 55.50 ± 3.50 kg, were enrolled in the study. From each animal, blood samples were collected before shearing (T_PRE_) and 5 (T_POST5_) and 60 (T_POST60_) minutes after the end of the shearing procedure in order to assess the values of hematological parameters, serum cortisol, total proteins and protein fractions, including albumin, α-, β1-, β2- and γ-globulins. According to statistical analysis results, albumin values were lower at T_POST60_ than T_POST5_ (*p* < 0.01), whereas α- and β2-globulins and the A/G ratio were higher at T_POST60_ with respect to T_PRE_ (*p* < 0.01) and T_POST5_ (*p* < 0.01). A higher serum concentration of cortisol was found at T_POST5_ and T_POST60_ than T_PRE_ (*p* < 0.01), and at T_POST60_ than T_POST5_ (*p* < 0.01). The serum cortisol values were negatively correlated with the serum values of albumin, β1-globulins and A/G ratio at T_POST60_, and positively correlated with α- and β2-globulins at T_POST5_ and T_POST60_. The decrease in the albumin concentration and the increase in the α- and β2-globulins observed in ewes after shearing with respect to the baseline values suggests an acute phase response in shorn ewes. Additionally, the correlation found between the serum cortisol concentration and the serum protein fractions confirmed the immunomodulatory effect of this hormone, emphasizing the linkage between the endocrine and immune systems during an acute stress condition.

## 1. Introduction

Farm animals are daily subjected to a wide range of abiotic stressors and management practices which can often cause stress to the animal with negative consequences on its health status and welfare [1,2]. Shearing, one practice of routine management procedures in European countries [3], is applied to harvest the sheep’s wool to ameliorate the resistance of sheep to high temperatures in summer and for hygienic reasons [4]. The shearing procedure represents short-term acute stress for animals [2,5] who develop behavioral, autonomic, endocrine and immune responses to maintain homeostasis [2,3,4,5,6,7,8,9,10,11]. Stress responses are related not only to the nature and the intensity of the stressful stimulus but also to individual response predispositions or temperament [6,7]. A deep knowledge of stress responses in farm animals allows practitioners to understand the welfare status of the animals and to create appropriate housing and husbandry conditions to improve production efficiency and product quality [8]. Blood is the tissue mostly investigated to assess the animal health status. Specifically, the evaluation of the hematological profile of an animal is considered a useful and sensitive index to screen physiological and pathological modifications [9]. Under acute stress conditions, the endocrine system is stimulated, and, consequently, an increase in corticosteroids occurs [10,11]. The rise of glucocorticoids in the bloodstream also indicates an inflammation state accompanied by a clear increase and/or decrease in a number of plasma proteins known as acute-phase globulins [12]. It is well-known that glucocorticoids play a crucial permissive role in the synthesis of acute-phase globulins. The acute-phase response is part of the first line of defense against injury and/or stressors as it falls within the non-specific immune system [13,14]. The acute-phase serum protein response could be pictured by the serum protein electrophoretic pattern [15]. An acute-phase response encompasses the increase in positive acute-phase proteins and the decrease in negative acute-phase proteins. Thus, different globulin fractions are not the only parameters to be considered in order to evaluate the severity of an acute-phase response [16,17]. The physiological adaptation of sheep to shearing has been extensively investigated in the field of hematochemical response as well as a thermal homeostasis alteration of the sheared animal’s thermoregulation [4,18,19,20,21,22,23,24,25,26,27,28]. However, the early stress response of sheep to shearing has been poorly investigated. To the best of the authors’ knowledge, no studies on the influence of this common management practice on serum protein fractions are available in the scientific literature so far. In view of the above consideration, the present study aimed to determine the influence of acute stress by shearing procedures on hematological parameters, serum cortisol concentrations and serum protein electrophoretic patterns in Comisana sheep.

## 2. Materials and Methods

### 2.1. Animals and Experimental Design

For the study, a flock of about 100 Comisana breed sheep was chosen during the mild-dry season of May–June in Sicily, Italy (40°41′ N; 14°26′ E, 9 m above sea level). The sheep are shorn once a year, in late spring, according to the traditional techniques of Sicilian sheep breeding. The animals of the chosen flock grazed on natural pasture were penned at night, when they received a concentrated commercial food supplement of 200 g per head (crude protein (CP) 20.4% and 12.5 MJ metabolizable energy (ME)/kg dry matter (DM)). The sheep had free access to water and hay (CP 11.1% and 7.2 MJ ME/kg DM). A total of 20 adult ewes (55.50 ± 3.50 kg, aged 3–4 years old) shorn in the previous year and familiar with the handling required for venipuncture were chosen from the flock enrolled in the study. The enrolled ewes were defined as clinically healthy with no evidence of disease and free from internal and external parasites.

The shearing was performed on 10 June 2021 (ambient temperature 28 ± 2 °C and relative humidity 64 ± 6%); the procedure was performed on all sheep on the same day starting at 7:00 a.m., and it took about 1 h and 50 min to complete the procedure on all ewes. Shearing was executed by hand using traditional shearing scissors in a 15 m × 10 m pen, and it lasted approximately 5 min for each sheep. At the end of the shearing procedure, the animals were released into an adjacent enclosure after being marked by a progressive number through an appropriate colored spray to identify them.

### 2.2. Sampling Procedures and Laboratory Analysis

From each sheep, blood was drawn by jugular venipuncture (needle gauge of 20G) and collected into one vacutainer tube containing EDTA and two tubes with clot activator (Terumo Corporation, Tokyo, Japan) before shearing (T_PRE_) and 5 (T_POST5_) and 60 (T_POST60_) min after the end of shearing. The time taken to collect blood from each animal was 40 s, whereas the time between blood sampling of different animals was approximately 10 min. On EDTA whole blood samples, the values of white blood cells (WBCs), red blood cells (RBCs), hematocrit (Hct), hemoglobin (Hb), mean corpuscular hemoglobin (MCH), mean corpuscular hemoglobin concentration, mean corpuscular volume (MCV) and platelets (PLTs) were assessed by means of an automated hematology analyzer (HeCo Vet C; SEAC, Florence, Italy). Samples from the first tube with a clot activator were allowed to clot for 20 min at room temperature and, thereafter, were centrifuged at 2325× *g* for 15 min. By visual inspection, all the obtained sera were non-hemolysed, and they were analyzed to assess the total protein concentration through a commercially available kit (Biosystems S.A., Barcelona, Spain; biuret method using bovine album as a protein standard at a concentration of 6.02 g/dL) by means of an automated ultraviolet (UV) spectrophotometer (Slim; SEAC, Florence, Italy). In order to evaluate serum protein fractions, an electrophoresis analysis was performed using an automated system (Selvet24, Seleo Engineering, Naples, Italy) according to the procedures described by the manufacturer. The protein fractions were divided into albumin, α-, β1-, β2-, and γ-globulins according to previous findings on ovine species [29,30]. Relative protein concentrations within each fraction were determined as the optical absorbance percentage; then, the absolute concentration (g/dL) and albumin/globulin ratio (A/G) were calculated using the total protein concentration. Samples from the second tube with a clot activator were allowed to clot overnight at 4 °C before centrifuging at 1000× *g* for 20 min at 2–8 °C. The obtained sera concentration of cortisol was evaluated using an ELISA kit specific for ovine species (Bovine/Sheep Cortisol ELISA kit, Elabscience Biotechnology Inc. Kampenhout, Belgium) through a microwell plate reader (Sirio, SEAC, Florence, Italy). Calibrators and samples were run in duplicate, and samples exhibited parallel displacement to the standard curve for both ELISA analyses. Both the intra- and the inter-assay coefficients of variation were <10%.

### 2.3. Statistical Analysis

All data were normally distributed (Kolmogorov–Smirnov test, *p* > 0.05), and one-way analysis of variance (ANOVA) for repeated measures was used to assess significant changes in hematological parameters, serum cortisol concentration and serum protein profile in ewes at the three time points herein considered (T_PRE_, T_POST5_ and T_POST60_). Bonferroni’s post hoc comparison was applied to the obtained significances. Pearson’s correlation analysis was applied to assess whether the serum cortisol concentration correlated with the values of serum total proteins and their fractions measured in ewes before shearing and 5 and 60 min after the end of shearing procedures. The degree of correlation between these parameters was investigated by means of a linear regression model (y = a + bx). *p*-values < 0.05 were considered statistically significant. The software Prism v. 9.00 (Graphpad Software Ltd., San Diego, CA, USA, 2020) was used to perform statistical analysis.

## 3. Results

The results are shown as mean values ± standard deviation (SD).

**Figure 1:**
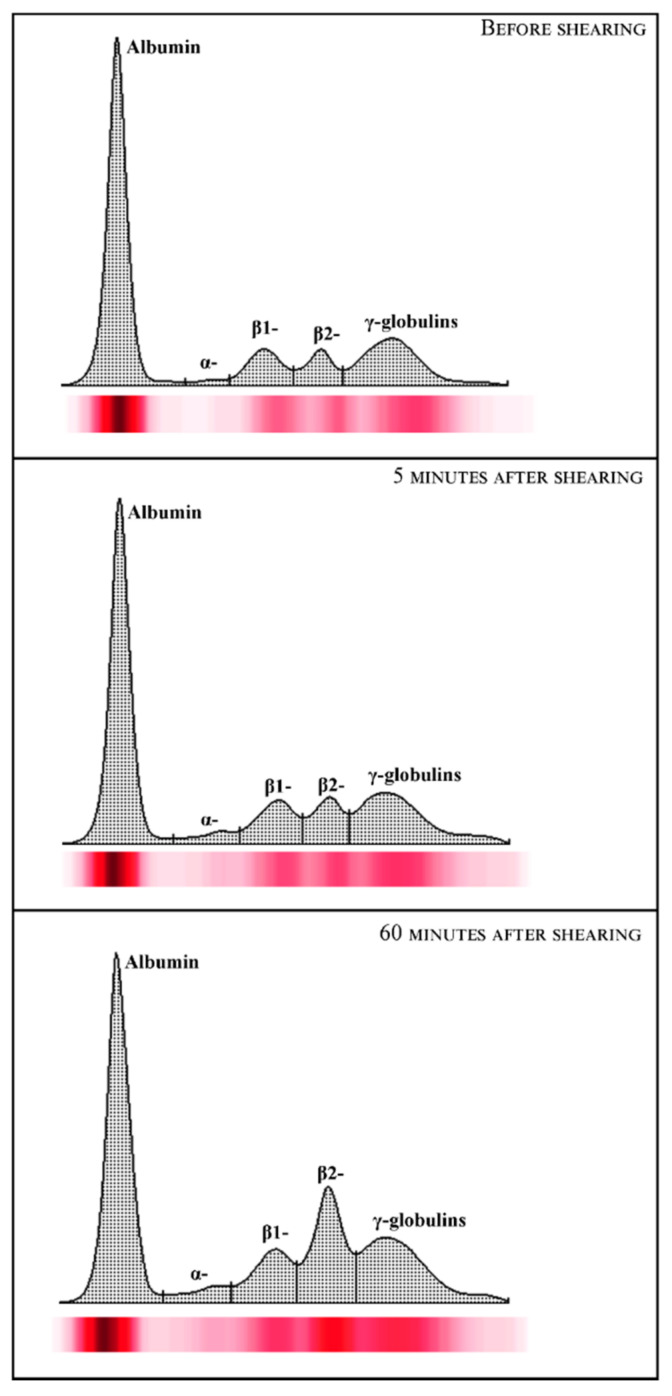
Representative cellulose acetate electrophoretograms of serum proteins were obtained from ewes before shearing (T_PRE_) and after 5 (T_POST5_) and 60 (T_POST60_) min from the end of the shearing procedure.

**Figure 2:**
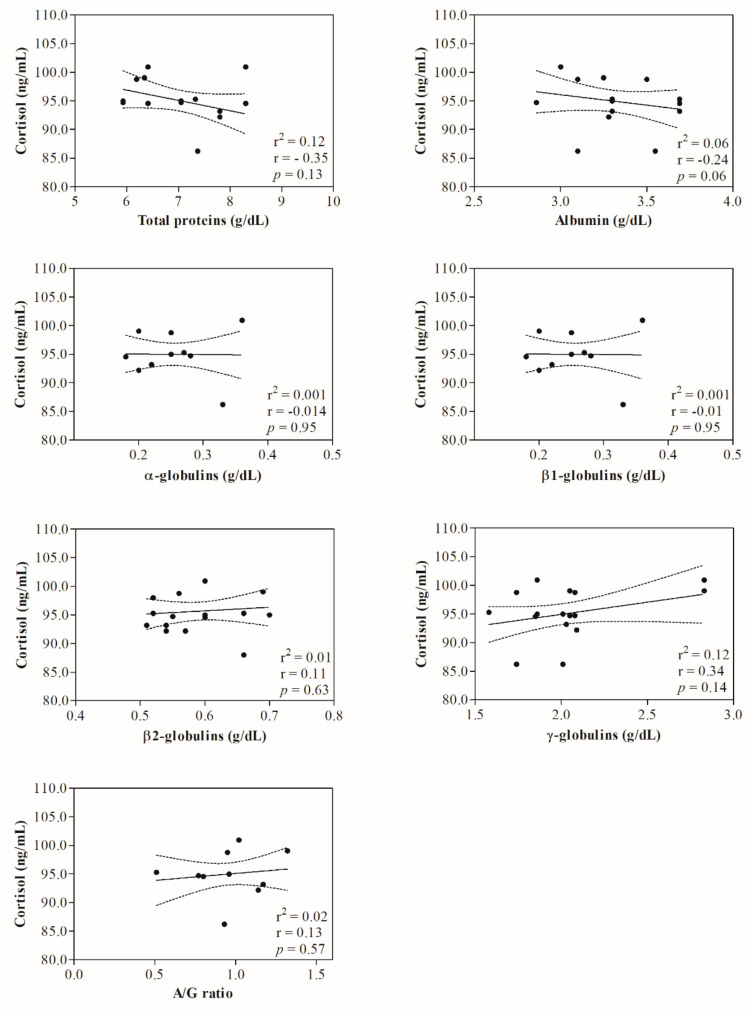
Linear regression model results found between the serum values of cortisol and the serum concentration of serum total proteins, albumin, α-, β1-, β2-, γ-globulins and the A/G ratio measured in ewes before the shearing procedure (T_PRE_).

**Figure 3:**
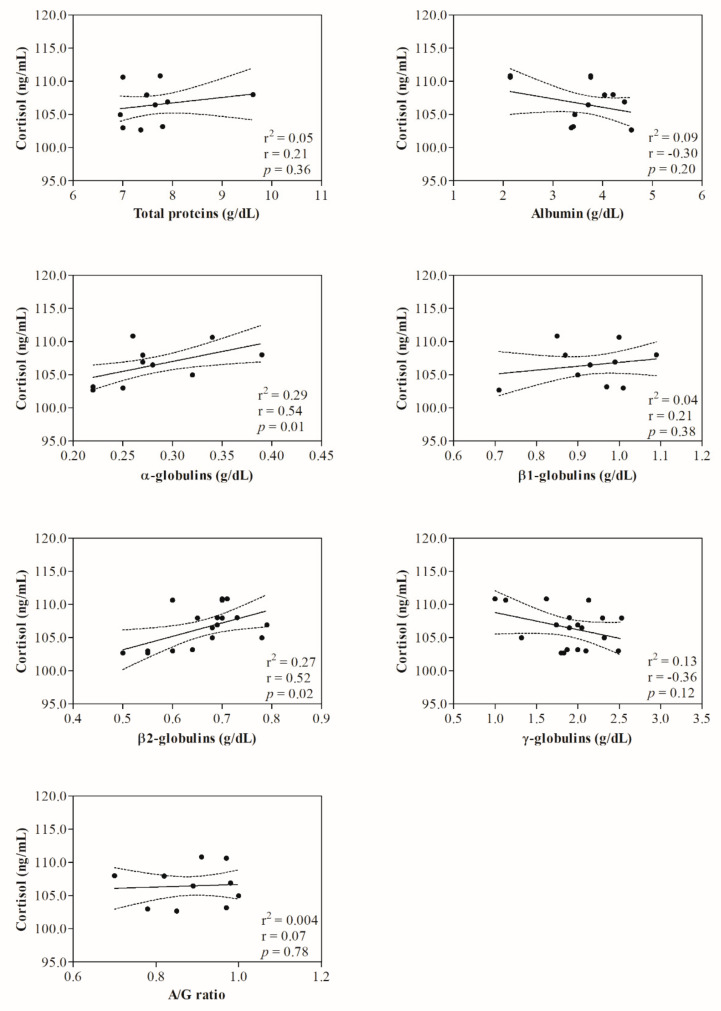
Linear regression model results found between the serum values of cortisol and the serum levels of total proteins, albumin, α-, β1-, β2-, γ-globulins and the A/G ratio measured in ewesafter 5 min from the end of the shearing procedure (T_POST5_).

**Figure 4:**
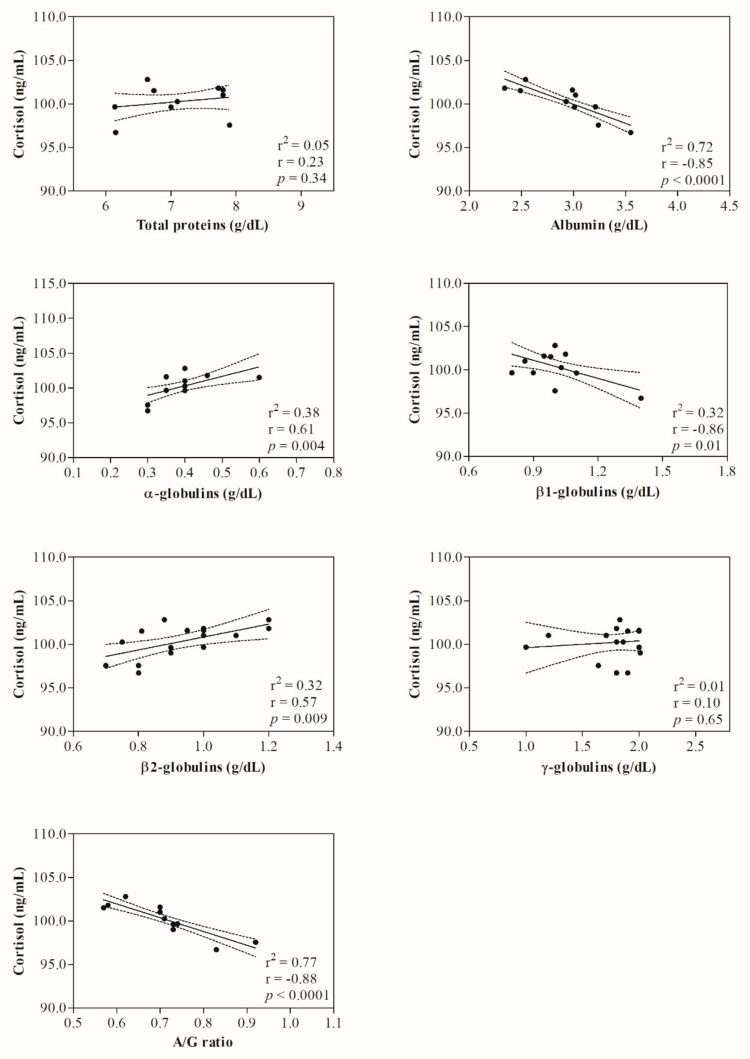
Linear regression model results found between the serum values of cortisol and the serum levels of serum total proteins, albumin, α-, β1-, β2-, γ-globulins and the A/G ratio measured in ewes after 60 min from the end of the shearing procedure (T_POST60_).

Statistical analysis of data showed no significant change (*p* > 0.05) in hematological parameters investigated in ewes following the shearing procedure (Table 1). The representative protein electrophoretograms obtained from serum samples of Comisana ewes before and after 5 and 60 min from the end of the shearing procedure are displayed in Figure 1. As reported in Table 2, albumin values were statistically significantly lower at T_POST60_ than T_POST5_ (*p* < 0.01); additionally, α- and β2-globulins and the A/G ratio were higher at T_POST60_ with respect to T_PRE_ (*p* < 0.01) and T_POST5_ (*p* < 0.01). No statistically significant changes were found in the serum concentration of total proteins (*p* > 0.05), β1- (*p* > 0.05) and γ-globulins (*p* > 0.05) throughout the experimental period. Higher serum concentrations of cortisol were found at T_POST5_ (*p* < 0.01) and T_POST60_ (*p* < 0.01) than T_PRE_, and at T_POST60_ (*p* < 0.01) than T_POST5_ (Table 2). No significant correlation was found between the serum cortisol concentration and the hematological parameters measured in investigated ewes before (T_PRE_) (*p* > 0.05) and after the shearing procedure (T_POST5_ and T_POST60_) (*p* > 0.05) (Table 3). The serum cortisol values were not correlated with the serum concentration of total proteins and γ-globulins measured in investigated ewes at T_PRE_ (*p* > 0.05), T_POST5_ (*p* > 0.05) and T_POST60_ (*p* > 0.05), whereas the concentration of this hormone was negatively correlated with the serum values of albumin (*p* < 0.05), β1-globulins (*p* < 0.05) and the A/G ratio (*p* < 0.05) at T_POST60_, and positively correlated with α- (*p* < 0.05) and β2-globulins (*p* < 0.05) at T_POST5_ and T_POST60_. The linear regression model confirmed the statistically significant correlations found among selected parameters (Figure 2, Figure 3 and Figure 4).

## 4. Discussion

Shearing includes several procedures, such as animal capture, separation, immobilization and tying, which are a potential source of acute stress.

It has been well established that acute stressors could lead to a transient increase in cells in the bloodstream as a consequence of splenic contraction following catecholamine stimulation as the so-called defence alarm reaction [31]. Under stress conditions, there is also a cortisol-mediated release of cells from the bone marrow [31]. According to the results obtained in the current study, hematological parameters did not show differences related to the shearing procedure in ewes. The missing change in hematological indices in stressed ewes could be due to the timing of blood sampling. In the current study, blood was collected before shearing and 5 and 60 min after the end of the stressor experience; hence, the transient increase of cells into the bloodstream could have been missed.

Contrariwise, it was found that shearing influenced some serum protein fractions measured in investigated ewes. The changes in total serum protein concentrations, as well as their fractional distribution and albumin/globulin ratio, are usually the first signs of homeostasis perturbation and/or disease conditions [32]. According to the serum protein electrophoretic pattern previously suggested for ovine species [29], five protein fractions were observed in sera obtained from ewes enrolled in the current study. 

The analysis of protein electrophoretograms obtained from serum samples of Comisana ewes showed that a decrease in the albumin concentration and an increase in the α- and β2-globulins were found in ewes after shearing. These findings suggest an acute phase response onset as an integral part of an acute stress response in ewes. The pro-inflammatory mediators, including cytokines, are the main factors involved in the regulation network of an acute-phase response [12] which stimulates the liver to synthesize some acute-phase proteins (APPs) whose blood levels increase. Thus, they are known as positive APPs. Other proteins, known as negative APPs, display decreasing trends in response to challenges [12]. The α- and β2-globulins include the main positive APPs (i.e., alpha 1-acid glycoprotein, caeruloplasmin, complement proteins, C-reactive protein, fibrinogen, haptoglobin, serum amyloid A). The β1-globulins fraction includes transferrin, which decreases in response to challenges like albumin. It is well known that the cortisol concentration rises in sheep subjected to handling and shearing as a stress response [22,27]. In agreement, the serum cortisol concentrations increased in ewes after shearing compared to pre-shearing, highlighting that this procedure would be stressful due to different factors linked to shearing, including handling of animals and isolation [22,33]. It is noteworthy that, in the current study, α- and β2-globulins were positively correlated with serum cortisol levels at T_POST5_ and T_POST60_, whereas albumin and β1-globulins showed a negative correlation with the serum concentration of this hormone at T_POST60_. Cortisol is known to have immunomodulatory effects and, together with catecholamines, affects the proliferation of B and T cells, cytokine production, and antibody production upon acute stress conditions [6,7].

Studies carried out on rats suggested that glucocorticoids and catecholamine could trigger the synthesis of proteins involved in the acute-phase response [34,35]. In agreement with this, the results obtained in the current study lead us to hypothesize that cortisol could be one of the mediators of mechanisms orchestrating the fine and complex acute phase response of the animal under stress circumstances. Additionally, the findings seem to reinforce the well-established idea that, upon a stressful condition, the immune and endocrine systems cross-talk to re-establish homeostasis of the organism [6].

## 5. Conclusions

According to the results herein gained, the acute stress caused by the shearing procedure induced a dynamic change in the electrophoretic serum protein pattern and the cortisol concentration. The electropherograms obtained following shearing suggest an acute-phase response as highlighted by the increase of α- and β-globulins, which are known to include many acute-phase proteins. Additionally, the significant relationship found between the serum concentration of cortisol, the albumin values, and α-, β1-, and β2-globulins confirmed the immunomodulatory effect of this hormone and emphasized the linkage between the endocrine and immune systems during acute stress.

## Figures and Tables

**Table 1:** White blood cells (WBCs), red blood cells (RBCs), hematocrit (Hct), hemoglobin (Hb), mean corpuscular volume (MCV), mean corpuscular hemoglobin (MCH), mean corpuscular hemoglobin concentration (MCHC) and platelets (PLTs) measured in ewes before shearing (T_PRE_) and after 5 and 60 min (T_POST5_ and T_POST60_) from the end of shearing. Data are expressed as mean values ± standard deviation (SD).

Hematological Parameters	Experimental Period		
	T_PRE_	T_POST5_	T_POST60_
WBCs (×10^3^/µL)	7.82 ± 1.58	7.48 ± 1.45	7.83 ± 1.61
RBCs (×10^6^/µL)	10.71 ± 0.78	10.50 ± 0.62	10.47 ± 0.81
Hct (%)	36.55 ± 4.72	35.81 ± 4.92	34.41 ± 3.98
Hb (g/dL)	9.78 ± 0.91	9.68 ± 1.05	9.59 ± 0.97
MCV (fL)	35.60 ± 5.14	35.62 ± 5.16	34.50 ± 5.20
MCH (pg)	9.51 ± 0.87	9.60 ± 0.90	9.57 ± 0.86
MCHC (%)	26.94 ± 0.40	27.19 ± 0.83	28.03 ± 1.78
PLTs (×10^3^/µL)	604.85 ± 168.48	636.08 ± 119.30	575.45 ± 101.08

**Table 2:** Serum cortisol, total proteins, albumin, α-, β1-, β2-, γ-globulins and the A/G ratio were measured in ewes before shearing (T_PRE_) and after 5 and 60 min (T_POST5_ and T_POST60_) from the end of the shearing procedure, together with statistically significant changes. Data are expressed as mean values ± standard deviation (SD).

Serum Parameters	Experimental Period		
	T_PRE_	T_POST5_	T_POST60_
Cortisol (ng/mL)	94.99 ± 4.14	100.3 ± 1.93 ^a^	106.5 ± 2.98 ^a,b^
Total proteins (d/dL)	7.05 ± 0.80	7.65 ± 0.78	7.10 ± 0.68
Albumin (g/dL)	3.30 ± 0.28	3.71 ± 0.68	2.93 ± 0.38 ^b^
α-globulins (g/dL)	0.26 ± 0.06	0.28 ± 0.05	0.40 ± 0.08 ^a,b^
β1-globulins (g/dL)	0.90 ± 0.11	0.93 ± 0.10	1.03 ± 0.14
β2-globulins (g/dL)	0.58 ± 0.06	0.68 ± 0.08	0.88 ± 0.10 ^a,b^
γ-globulins (g/dL)	2.01 ± 0.33	2.05 ± 0.31	1.85 ± 0.13
A/G ratio	0.89 ± 0.10	0.96 ± 0.23	0.71 ± 0.11 ^b^

Note: statistical significances: ^a^ versus T_PRE_ (*p* < 0.01); ^b^ versus T_POST5_ (*p* < 0.01).

**Table 3:** Coefficients of correlation (Pearson’s r and *p*-value) among the serum concentration of cortisol, the levels of white blood cells (WBCs), red blood cells (RBCs), hematocrit (Hct), hemoglobin (Hb), mean corpuscular volume (MCV), mean corpuscular hemoglobin (MCH), mean corpuscular hemoglobin concentration (MCHC) and platelets (PLTs) measured in ewes before shearing (T_PRE_) and after 5 and 60 min (T_POST5_ and T_POST60_) from the end of shearing. *p*-values < 0.05 were considered statistically significant.

		WBCs (×10^3^/µL)	RBCs (×10^6^/µL)	Hct (%)	Hb (g/dL)	MCV (fL)	MCH (pg)	MCHC (%)	PLTs (×10^3^/µL)
Cortisol (ng/mL)	T_PRE_	r = 0.24	r = −0.99	r = −0.02	r = −0.20	r = −0.10	r = −0.30	r = −0.13	r = −0.20
		*p* = 0.31	*p* = 0.70	*p* = 0.95	*p* = 0.40	*p* = 0.67	*p* = 0.20	*p* = 0.59	*p* = 0.40
	T_POST5_	r = −0.44	r = −0.37	r = 0.18	r = 0.10	r = 0.31	r = 0.43	r = 0.12	r = −0.29
		*p* = 0.06	*p* = 0.11	*p* = 0.46	*p* = 0.68	*p* = 0.18	*p* = 0.06	*p* = 0.61	*p* = 0.21
	T_POST60_	r = −0.24	r = 0.27	r = −0.44	r = −0.15	r = −0.16	r = 0.13	r = 0.06	r = −0.26
		*p* = 0.30	*p* = 0.26	*p* = 0.06	*p* = 0.54	*p* = 0.50	*p* = 0.61	*p* = 0.81	*p* = 0.27

## Data Availability

The data presented in this study are available on request from the corresponding author.
